# Opportunistic lung cancer screening with low‐dose computed tomography in National Cancer Center of China: The first 14 years' experience

**DOI:** 10.1002/cam4.6914

**Published:** 2024-01-17

**Authors:** Wei Tang, Li Liu, Yao Huang, Shijun Zhao, Jianwei Wang, Min Liang, Yujing Jin, Lina Zhou, Ying Liu, Yanyan Tang, Zhijian Xu, Kai Zhang, Fengwei Tan, Nan Bi, Zhijie Wang, Fei Wang, Ni Li, Ning Wu

**Affiliations:** ^1^ Department of Diagnostic Radiology, National Cancer Center/National Clinical Research Center for Cancer/Cancer Hospital Chinese Academy of Medical Sciences and Peking Union Medical College Beijing China; ^2^ Radiology Department Beijing Chaoyang Hospital, Capital Medical University Beijing China; ^3^ Department Nuclear Medicine (PET‐CT Center), National Cancer Center/National Clinical Research Center for Cancer/Cancer Hospital Chinese Academy of Medical Sciences and Peking Union Medical College Beijing China; ^4^ Department of Cancer Prevention, National Cancer Center/National Clinical Research Center for Cancer/Cancer Hospital Chinese Academy of Medical Sciences and Peking Union Medical College Beijing China; ^5^ Department of Thoracic Surgery, National Cancer Center/National Clinical Research Center for Cancer/Cancer Hospital Chinese Academy of Medical Sciences and Peking Union Medical College Beijing China; ^6^ Department of Radiation Oncology, National Cancer Center/National Clinical Research Center for Cancer/Cancer Hospital Chinese Academy of Medical Sciences and Peking Union Medical College Beijing China; ^7^ CAMS Key Laboratory of Translational Research on Lung Cancer, State Key Laboratory of Molecular Oncology, Department of Medical Oncology, National Cancer Center/National Clinical Research Center for Cancer/Cancer Hospital Chinese Academy of Medical Sciences and Peking Union Medical College Beijing China; ^8^ Office of Cancer Screening, National Cancer Center/National Clinical Research Center for Cancer/Cancer Hospital Chinese Academy of Medical Sciences and Peking Union Medical College Beijing China

**Keywords:** low‐dose computed tomography, lung cancer, nodule, screening

## Abstract

**Background:**

In China, over 50% of lung cancer cases occur in nonsmokers. Thus, identifying high‐risk individuals for targeted lung cancer screening is crucial. Beyond age and smoking, determining other risk factors for lung cancer in the Asian population has become a focal point of research. Using 30,000 participants in the prospectively enrolled cohort at China's National Cancer Center (NCC) over the past 14 years, we categorized participants by risk, with an emphasis on nonsmoking females.

**Materials and Method**s**:**

Between November 2005 and December 2019, 31,431 individuals voluntarily underwent low‐dose computed tomography (LDCT) scans for lung cancer screening at the NCC. We recorded details like smoking history, exposure to hazards, and family history of malignant tumors. Using the 2019 NCCN criteria, participants were categorized into high‐, moderate‐, and low‐risk groups. Additionally, we separated non‐high‐risk groups into female never smokers (aged over 40) exposed to second‐hand smoke (SHS) and others. Any positive results from initial scans were monitored per the I‐ELCAP protocol (2006), and suspected malignancies were addressed through collaborative decisions between patients and physicians. We analyzed and compared the detection rates of positive results, confirmed lung cancers, and cancer stages across risk, age, and gender groups.

**Results:**

Out of 31,431 participants (55.9% male, 44.1% female), 3695 (11.8%) showed positive baseline LDCT scans with 197 (0.6%; 106 females, 91 males) confirmed as lung cancer cases pathologically. Malignancy rate by age was 0.1% among those aged under 40 years, 0.4% among those aged 40–49 years, 0.8% among those aged 50–59 years, and 1.2% among those aged 60 years and older. From the 25,763 participants (56.9% male, 43.1% female) who completed questionnaires, 1877 (7.3%) were categorized as high risk, 6500 (25.2%) as moderate risk, and 17,386 (67.5%) as low risk. Of the 23,886 in the non‐high‐risk category, 8041 (33.7%) were females over 40 years old exposed to SHS. The high‐risk group showed the highest lung cancer detection rate at 1.4%. However, females exposed to SHS had a notably higher detection rate than the rest of the non‐high‐risk group (1.1% vs. 0.5%; *p* < 0.0001). In this cohort, 84.8% of the detected lung cancers were at an early stage.

**Conclusions:**

In our study, using LDCT for lung cancer screening proved significant for high‐risk individuals. For non‐high‐risk populations, LDCT screening could be considered for nonsmoking women with exposure to SHS.

## 1 INTRODUCTION

Lung cancer represents a significant global public health challenge due to its high prevalence, substantial treatment costs, and a dismal 5‐year survival rate that hovers between 10% and 20% in most countries.^1^, ^2^, ^3^, ^4^ In 2020, an estimated 2.2 million new cases of lung cancer were found, in addition to 1.8 million fatalities, making it as the second most commonly diagnosed cancer and the foremost cause of cancer‐related deaths worldwide.^5^ In China, lung cancer mortality has surged approximately 7‐fold from the 1970s to 2015. Projections suggest that by 2030, the number of lung cancer‐related deaths may reach or exceed 850,000.^6^, ^7^, ^8^

Numerous international randomized controlled trials (RCT) have confirmed that screening high‐risk individuals using low‐dose CT can significantly reduce the mortality of lung cancers.^9^, ^10^, ^11^, ^12^, ^13^, ^14^, ^15^ However, the fact that these RCTs were undertaken in selective individuals who were over 50 years old with a smoking history of more than 20 pack‐years and thus many existing lung screening guidelines based on the RCTs could miss about 50% of lung cancers, especially of those in light or never smokers who need to be paid attention to.^5^, ^9^, ^16^ Otherwise, these guidelines may not be entirely fitting for Asian populations. Notably, in Asia, over 40% of lung cancer cases are unrelated to tobacco smoking,^16^, ^17^, ^18^ a stark contrast to approximately 10%–15% found in Europe and North America.^19^ For instance, a study conducted in Nagano prefecture found that 51.7% of all lung cancers were detected in individuals who never smoked.^20^ Another research in Hitachi city reported a slightly higher figure, with 57.5% of lung cancers identified in nonsmokers.^21^ In Japan, it is alarming to note that over half of the lung cancers detected in screening programs were among nonsmokers.^16^ This is further emphasized by the finding that lung cancer‐related deaths in nonsmokers rank as the fifth most common cause of death in men and the third in women.^22^ These statistics reflect a significantly high prevalence of lung cancers unrelated to smoking, accounting for 31% in men and a staggering 80% in women.^22^ Similarly, in China, approximately 50% of lung cancer mortality in men and 6% in women were attributable to active smoking.^23^ Therefore, how to effectively and precisely target high‐risk individuals for lung cancer to enhance the benefits of lung cancer screening, and in addition to age and smoking, what other risk factors for lung cancer exist in the Asian population have become hot topics of interest in lung cancer screening.

Opportunistic lung cancer screening takes place outside of an organized, systematic screening program and results from health‐seeking behavior of patients. This presents an ideal opportunity for us to explore the potential effect of screening across diverse subgroups, particularly in areas where evidence from expansive screening programs remains nascent or is yet to be solidified. The Cancer Hospital of the Chinese Academy of Medical Sciences and National Cancer Center of China (NCC) initiated practices on LDCT lung cancer screening in 2005 and formally became a member of the International Early Lung Cancer Screening Action Program (I‐ELCAP) in 2007. Based on the core principles of I‐ELCAP, we have been carrying out lung cancer screenings. This study reviewed our experiences with more than 30,000 participants over the past 14 years. Utilizing hospital‐based opportunistic low‐dose screening, we stratified participants based on risk levels to better identify individuals at high risk for lung cancer, especially nonsmoking females. The insights garnered would offer beneficial references for China and other regions with similar scenarios, contributing to the existing knowledge on the subject.

## 2 METHODS

### 2.1 Study population

From November 2005 to December 2019, asymptomatic healthy people that willingly underwent LDCT lung cancer screening in NCC were prospectively enrolled in this study. All attendees received counseling and participated in shared decision‐making before screening. This study was approved by the Ethics Committee of National Cancer Center of China, Cancer Hospital Chinese Academy of Medical Science, Beijing, China (No. KS1407). Consent was obtained from every participant in the current study.

### 2.2 Data acquisition

Participants were invited to complete standardized questionnaires including demographic information (name, gender, date of birth, height, weight, occupation, education level, and contact information), smoking history, second‐hand smoke (SHS) exposure, asbestos or other hazard exposure, as well as family history of lung cancer, when registered at the Department of the Cancer Prevention. SHS means that individuals have lived or worked in the same room with smokers.

Participants were classified into three groups according to the National Comprehensive Cancer Network (NCCN version 2019) guideline. The high‐risk group includes individuals aged 55–74 years with a smoking history of over 30 pack‐years (PY), or aged over 50 years with a smoking history of over 20 PY and had at least one additional lung cancer risk factor (i.e., family history of lung cancer in first‐degree relatives, occupational exposure, and radon exposure). Moderate‐risk group includes those who aged 50 years or older and with a 20 or more pack‐year history of smoking or SHS exposure but no additional lung cancer risk factors. Low‐risk group includes those younger than 50 years and/or with a smoking history less than 20 PY. Furthermore, due to the distinctive characteristics of nonsmoking females, we classified our participants into the high‐risk group (NCCN 2019), the group of females aged over 40 years who have never smoked but have been exposed to SHS, and the remaining non‐high‐risk group.

### 2.3 LDCT scan and follow‐up procedures

The LDCT scan protocol and nodule management largely follow the I‐ELCAP schedule (I‐ELCAP Screening Protocol 2006).^24^ The detailed information regarding LDCT parameters, imaging evaluation and management of nodules were available in the supplemental documents. Positive findings were defined as any solid or partially solid nodule with a diameter ≥5 mm or a nonsolid nodule with a diameter ≥8 mm. Semi‐positive findings were defined as noncalcified nodules with diameter smaller than the above criteria. Emphysema and coronary artery calcification were also evaluated and recorded.

### 2.4 Outcome ascertainment

The present study exclusively considered lung cancers detected at baseline. For individuals who underwent surgery during the initial screening, tissue samples or cytology specimens obtained from surgical resection, percutaneous pulmonary biopsy, bronchoscopy, thoracoscopic biopsy, or pleural effusion cytology examination were used for histologic analysis, depending on the specific circumstances. In instances of pathologically confirmed lung cancer, comprehensive data including diagnostic procedures, histologic type, tumor stage, initial treatment, and postoperative care were meticulously compiled from certified medical records.

Histopathological assessments and tumor staging adhered to the 2015 World Health Organization (WHO) classification of lung tumors^25^ and the eighth edition of the TNM Classification for lung cancer.^26^ Early‐stage lung cancers were defined as Stage 0‐I.

### 2.5 Statistical analysis

The characteristics of the study population were analyzed descriptively using variables such as age at recruitment, sex, body mass index (BMI), exposure to asbestos or other hazards, and family history of lung cancer. Quantitative variables were presented as either mean ± standard deviation (SD) or median (interquartile range [IQR]), depending on their distribution. Categorical variables were expressed in terms of frequency (N) and percentage (%). To test differences between groups in demographic and risk factors, we employed chi‐squared tests for categorical variables (using Fisher's tests where appropriate) and the Mann–Whitney test for continuous variables. Analyses were conducted using R software (R version 3.4.1).

## 3 RESULTS

From November 2005 to December 2019, 31,431 (male 55.9%, female 44.1%) participants underwent the LDCT lung cancer screening. Among them, 3695 (11.8%) individuals were identified with positive results on the baseline scan; and 197 (0.6%) participants (male 46.2%, female 53.8%) were pathologically proved with lung cancer at baseline. The age‐specific lung cancer detection rates among all participants were calculated; not surprisingly, the highest rate of 1.2% was noted in individuals older than 60 years old. (Table 1).

**TABLE 1:** LDCT findings by age groups of 31,431 participants.

Characters	Age at enrollment (*n*, %)				*p*
	<40 yrs. (4050, 12.9)	40–49 yrs. (11,710, 37.3)	50–59 yrs. (10,133, 32.2)	≥60 yrs. (5538, 17.6)	
Gender (*n*, %)					<0.0001
Male (17,567, 55.9)	2655 (65.6)	6511 (55.6)	5512 (54.4)	2889 (52.2)	
Female (13,864, 44.1)	1395 (34.4)	5199 (44.4)	4621 (45.6)	2649 (47.8)	
Baseline results of LDCT (*n*, %)					<0.0001
Negative (10,343, 32.9)	1840 (45.4)	4220 (36.0)	3018 (29.8)	1265 (22.8)	
Semi‐positive (17,393, 55.3)	1972 (48.7)	6477 (55.3)	5775 (60.0)	3169 (57.2)	
Positive (3695, 11.8)	238 (5.9)	1013 (8.7)	1340 (13.2)	1104 (19.9)	
Cancer cases (197, 0.6)	4 (0.1)	49 (0.4)	77 (0.8)	67 (1.2)	
Other findings on LDCT (*n*, %)					<0.0001
Calcified pulmonary nodules (10,033, 31.9)	887 (21.9)	3318 (28.3)	3390 (33.5)	2438 (44.0)	
Emphysema (1238, 3.9)	37 (1.0)	226 (1.9)	487 (4.8)	488 (8.8)	
Coronary artery calcification (4436, 14.1%)	65 (1.6)	650 (5.6)	1693 (16.7)	2028 (36.6)	

Among all participants, 25,763 attendees (male 56.9%, female 43.1%, with a median age of 49 years.) completed the questionnaires. Based on the reported information and the NCCN (2019) criteria, 1877 (7.3%) were included into the high‐risk group, 6500 (25.2%) into moderate‐risk group, and 17,386 (67.5%) into low‐risk group, as the sex and age distribution presented on Table 2. Smoking rate was about 32.0% (25.3% for current smokers and 6.7% for former smokers) in this cohort. SHS exposure was common (82.5%) among all participants regardless of gender. In total, 1284 (5.0%) participants had a history of asbestos exposure and 4332 (16.8%) had family history of lung cancer (Table 2). Except for the high‐risk group, female never smokers with SHS whose age were over 40 years and other non‐high‐risk group were 8041 (31.2%) and 15,845 (61.5%), respectively (Table 3).

**TABLE 2:** Baseline characteristics of the study population across different risk groups (25,763 participants).

	NCCN groups (*n*, %)			*p*
	High‐risk group (1877, 7.3)	Moderate‐risk group (6500, 25.2)	Low‐risk group (17,386, 67.5)	
Gender (*n*, %)				<0.0001
Male (14,661, 56.9)	1795 (95.6)	3278 (50.4)	9588 (55.1)	
Female (11,102, 43.1)	82 (4.4)	3222 (49.6)	7798 (44.9)	
Age (mean ± SD, min–‐max)	59.0 ± 5.4 (50–74)	57.3 ± 6.5 (50–94)	45.9 ± 8.6 (20–90)	<0.0001
BMI (mean ± SD, min–max)	25.6 ± 4.9 (15.8–33.3)	25.0 ± 4.9 (15.6–38.9)	24.9 ± 6.5 (15.6–39.0)	=0.107
Smoking status (*n*, %)				
Current smoker (6524, 25.3)	1616 (86.1)	1227 (18.9)	3681 (21.2)	
Former smoker (1718, 6.7)	261(13.9)	544 (8.4)	913 (5.2)	
Smoking Index (median, min–max)	37.9 (20–180)	21.6 (0.1–120)	17.9 (0–120)	<0.0001
Never smoker (17,521, 68.0)	—	4729 (72.8)	12,792 (73.6)	
SHS exposure (21,261, 82.5)	1747 (92.7)	6408 (98.6)	13,106 (75.4)	<0.0001
Asbestos exposure history (1284, 5.0)	268 (14.2)	0 (0)	1016 (5.8)	<0.0001
Lung cancer family history (4332, 16.8)	535 (28.4)	0 (0)	3797 (21.8)	<0.0001

**TABLE 3:** Lung cancer stage distribution by different groups (25,763 participants).

	NCCN group (*n*, %)			*p*
	High‐risk group (1877, 7.3)	Female with SHS over 40 years. (8041, 31.2)	Other non‐high‐risk group (15,845, 61.5)	
Results of baseline LDCT				<0.0001
Negative (8588, 33.3)	521 (27.8)	2498 (31.1)	5569 (35.1)	
Semi‐positive (14,185, 55.1)	984 (52.4)	4608 (57.3)	8593 (54.2)	
Positive (2990,11.6)	372 (19.8)	935 (11.6)	1683 (10.7)	
Lung cancer (186^a^, 0.7)	26 (1.4)	87 (1.1)	73 (0.5)	
Stage of lung cancer^b^				<0.0001
Stage 0‐I (140, 84.8%)	16 (76.2)	70 (90.9)	54 (80.6)	
Stage II (6, 3.6%)	1 (3.8)	1 (1.3)	4 (6.0)	
Stage III (12, 6.6%)	4 (19.0)	3 (3.9)	5 (7.4)	
Stage IV (7, 4.2%)	0 (0)	3 (3.9)	4 (6.0)	

^a^ 11 lung cancer patients did not belong to any group without the complete questionnaire; 95 female among 186 cases.

^b^ 32 lung cancer patients underwent treatment in other hospital without exactly tumor stage.

A total of 197 lung cancer cases were confirmed by pathology (90 male, 107 female; median age: 55 years, [IQR: 38–80] years). Eleven cases were diagnosed as malignancies without accurate pathological types in other hospitals. In total, 186 lung cancer patients were confirmed by pathology and the lung cancer detection rate of female with SHS over 40 years was lower than the high‐risk group (1.1% vs. 1.4%), but much higher than other non‐high‐risk group (1.1% vs. 0.5%, *p* < 0.0001). In 165 patients with definite clinical staging (140 at Stage 0‐I, 6 at Stage II, 12 at Stage III, and 7 at Stage IV), the general proportion of early stage lung cancer was 84.8% (140 out of 165). The early lung cancer detection rate among female with SHS exposure group was higher than other non‐high‐risk group and high‐risk group (90.9% vs. 80.6% and 76.2%, *p* < 0.0001). Advanced lung cancer (Stage III–IV) accounts for 13.4% (9 out of 67) of patients in other non‐high‐risk group, which was higher (*p* < 0.0001) than the group of female with SHS over 40 years (6 out of 77, 7.8%), but significantly lower than the high‐risk group (4 out of 21, 19.0%) (Table 3).

**FIGURE 1:**
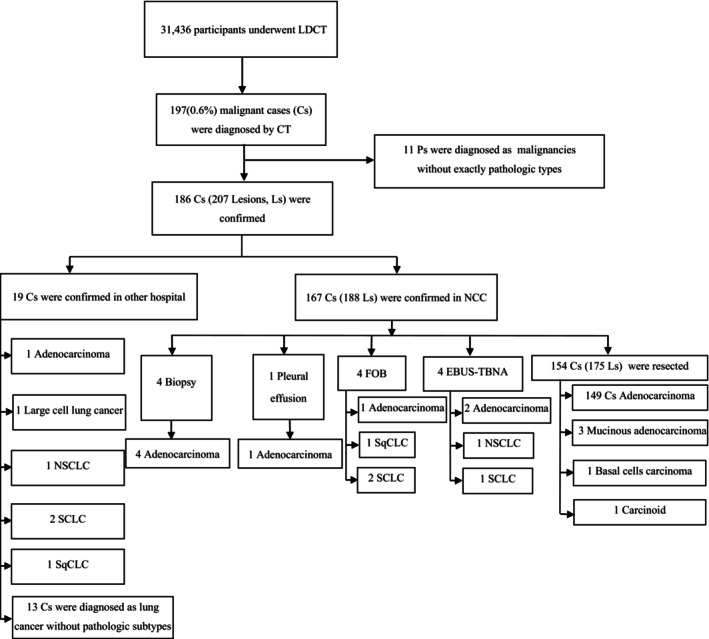
Flowchart of lung cancer diagnosis in the NCC cohort.

Totally 207 lesions got pathological diagnosis of lung cancer on 186 patients and 13 lesions on 11 patients were diagnosed as “lung cancer” without precise tumor information in other hospitals. Otherwise, 158 cases (158 out of 173, 91.3%) of adenocarcinoma, 5 cases of small cells lung cancer, 3 cases of mucinous adenocarcinoma, 2 cases of squamous cells lung cancer, 2 cases of non‐small cells lung cancer, 1 large cell lung cancer, 1 basal cell lung cancer, and 1 carcinoid cancer were confirmed by pathology (Figure 1).

In all participants, the detection rates of emphysema and coronary artery calcification were 3.9% and 14.1%, respectively. The data were comparable with the whole cohort, and an increasing tendency was noted along with the increase in age (Table 1).

## 4 DISCUSSION

Screening using LDCT has been demonstrated to significantly reduce the risk of lung cancer‐related death in heavy smokers.^27^ However, present screening guidelines and eligibility criteria overlook approximately 50% of lung cancers, predominantly in light smokers and those who have never smoked.^28^ The situation is notably pronounced in China, where between 30% and 60% of lung cancer patients have never smoked. This figure escalates in females, exceeding 90%.^29^, ^30^, ^31^ There is an evident need for a tailored LDCT screening approach for light smokers and nonsmokers to enhance individual outcomes and reduce the public health implications of lung cancer. As a long‐term study with a large sample size, opportunistic screening, in contrast to randomized controlled trials, offers insights that can help refine inclusion criteria, especially for nonsmoking Asian female populations.

In our opportunistic screening study, the lung cancer detection rate displays a correlation with age. The detection rates were 0.1% for those under 40 years, 0.4% for the 40–49 age group, 0.8% for 50–59 years, and 1.2% for individuals over 60 years. These findings suggest that older individuals are more likely to be diagnosed with lung cancer through the screening process.

The Asian population exhibits distinct differences from Caucasians with regard to lung cancer in several respects. Beyond the influence of tobacco smoking, pulmonary diseases and genetic predispositions pose significant risks within the Asian demographics. In China, approximately 20%–40% of the general population harbors mutations related to lung cancer, with the EGFR mutation present in 40%–50% of lung cancer patients.^32^, ^33^, ^34^, ^35^ These percentages are notably higher than those observed in the United States and Europe.^28^ Additionally, second‐hand smoke (SHS) and other hazardous exposures further amplify the lung cancer risk. Among never‐smoking lung cancer patients, exposure to SHS carries an odds ratio (OR) of 1.26, while household cooking fumes present an OR ranging from 1.22 (in Europe) to 2.12 (in China).^36^ It is important to note that in the current study, we could not assess the impact of cooking fumes on lung cancer incidence occurrence among nonsmoking individuals due to a lack of quantitative data. Subsequent research endeavors are necessary to quantify the specific effects within this subgroup. Polycyclic aromatic hydrocarbons, predominantly produced during cooking, have an OR of 1.40.^36^ A specific study highlighted a correlation between family history and the extent of cooking exposure in relation to lung cancer risk among nonsmoking Chinese females.^37^ This underscores the combined influence of genetic and environmental factors in lung cancer progression. Attributable to these differences, guidelines on lung cancer screening developed in the Western countries should not be directly applied to the Asian population. Consequently, 68.0% of the participants in this opportunistic screening study were never smokers. Although the lung cancer detection rate among low‐risk individuals stood at a modest 0.7%, a pronounced rate of 1.1% was observed among nonsmoking women over 40 years of age exposed to second‐hand smoke (SHS), in contrast to the 0.5% in other traditionally low‐risk demographics. This aligns with findings from studies in Asian countries, where nonsmoking females have been identified as having a heightened risk of lung cancer.^38^ As a result, these findings distinguish nonsmoking females as a unique subgroup when compared to other low‐risk individuals as defined by the USPSTF criteria.^39^ Our research offers valuable perspectives on broadening lung cancer screening criteria to encompass nonsmoking females, potentially refining the inclusion parameters.

Within this cohort, adenocarcinoma emerges as the predominant histological type of lung cancer, accounting for 91.3% (158 out of 173) of cases, and it is especially prevalent among women with an incidence of 97.9% (93 out of 95). This trend aligns with findings from other screening trials and clinical observations.^10^, ^12^, ^34^, ^35^, ^36^, ^37^ Over recent decades, there has been a marked increase in adenocarcinoma incidence among young East Asian women and nonsmokers.^40^, ^41^, ^42^ Some research attributes this rise to advancements in cigarette manufacturing processes, SHS exposure, and exposure to kitchen fuel fumes, linking them closely to the development of adenocarcinoma.^41^, ^43^, ^44^

The rate of early‐stage lung cancer diagnosis in our study stood at 84.8% (140 out of 165), surpassing rates reported in the NLST (54.8%)^45^ and NELSON (69%) studies.^46^ This disparity is likely attributable to our cohort's younger age distribution (with 51.4% under 50 years) and a higher proportion of non‐high‐risk participants (92.7%), in comparison with the NLST and NELSON cohorts. In the high‐risk category, 76.2% of patients were diagnosed at an early stage, figures that resonate with NLST (54.8%)^27^ and NELSON (69%)^46^ results. Such variations underscore the potential merits and logic behind LDCT screening in non‐high‐risk populations. Furthermore, LDCT screening extends its utility beyond cancer detection, offering insights into personal health metrics. Our study observed a heightened detection of conditions like emphysema and coronary artery calcification, both of which exhibited a correlation with advancing age.

### 4.1 Limitations

Our research has several limitations. First, the screening program being conducted in a single institution might introduce selection bias. Second, the self‐referral approach combined with self‐financed expenses tends to attract more health‐conscious individuals, further contributing to potential selection bias. Third, the underestimation of the lung cancer detection rate can be attributed to the fact that not all individuals with positive baseline LDCT results adhered to our recommendations. Finally, false‐negative cases of lung cancer could not be accounted for in our analysis due to a lack of access to this information. Future investigations should encompass a more comprehensive assessment of the risk of lung cancer within various subgroups, incorporating the examination of false‐negative cases.

## 5 SUMMARY

Through our extended experience, we have developed a comprehensive schedule that encompasses participant recruitment, standardized scanning, image interpretation, and nodule management within LDCT screening. A critical objective within these procedures is to identify an appropriate target population that aligns with local circumstances. Our study validated that LDCT screening was particularly beneficial for individuals at high to moderate risk. Although lung cancer detection rate was high among nonsmoking women exposed to SHS in the current study, more data are required to support the inclusion of this subgroup to the screening candidates. Personalized and precise screening strategies are the direction of future development.

## AUTHOR CONTRIBUTIONS

**Wei Tang:** Conceptualization (supporting); data curation (lead); formal analysis (equal); funding acquisition (supporting); investigation (equal); methodology (equal); resources (lead); validation (lead); visualization (lead); writing – original draft (lead); writing – review and editing (supporting). **Li Liu:** Methodology (supporting); validation (equal); visualization (supporting); writing – original draft (equal). **Yao Huang:** Conceptualization (supporting); investigation (equal); methodology (equal); project administration (supporting); supervision (supporting); writing – original draft (supporting). **Shijun Zhao:** Methodology (supporting). **Jianwei Wang:** Funding acquisition (supporting); investigation (equal); methodology (equal); project administration (supporting). **Min Liang:** Writing – review and editing (equal). **Yujing Jin:** Writing – original draft (supporting). **Lina Zhou:** Methodology (supporting). **Ying Liu:** Methodology (supporting). **Yanyan Tang:** Writing – review and editing (equal). **Zhijian Xu:** Methodology (supporting). **Kai Zhang:** Methodology (supporting). **Fengwei Tan:** Investigation (supporting). **Nan Bi:** Investigation (supporting). **zhijie wang:** Methodology (supporting). **Fei Wang:** Writing – review and editing (equal). **Ni Li:** Formal analysis (equal). **Ning Wu:** Conceptualization (lead); funding acquisition (lead); investigation (lead); methodology (lead); project administration (lead); supervision (lead); writing – review and editing (equal).

## FUNDING INFORMATION

This work was supported by the National Key R&D Program of China (2017YFC1308700, 2020AAA0109504), Beijing Hope Run Special Fund of Cancer Foundation of China (LC2021A25), Beijing Nova Program (Z201100006820070), National Natural Science Foundation of China (82273722, 81971616, 82204143), Beijing Natural Science Foundation (7222148), and CAMS Innovation Fund for Medical Sciences (CIFMS, 2021‐I2M‐C&T‐B‐065).

## Supporting information


Data S1.

## Data Availability

The analyzed datasets generated during the study are available from the corresponding author on reasonable request. Data collected for the study will not be made available to others.
