# Supplementary material for: Opportunistic lung cancer screening with low‐dose computed tomography in National Cancer Center of China: The first 14 years' experience
Source: Cancer Med. 2024 Jan 17;13(3):e6914. doi: 10.1002/cam4.6914 (PMC10904962; doi:10.1002/cam4.6914)
Supplement: Supplementary file 1 — Data S1. [file CAM4-13-e6914-s001.docx]

**Supplementary documents**

**LDCT parameters**

All the participants underwent LDCT scanning were performed on scanners from different manufacturers (GE LightSpeed 16 Slice CT scanner, GE LightSpeed VCT 64 slice CT scanner, GE Discovery 750 HD 64 slice CT scanner, GE Optima CT660 64 slice CT scanner, GE Revolution 256 slice CT scanner and Toshiba Aquilion one 64 slice CT scanner, from lung apex to base without contrast enhancement. Subjects were placed in the supine position with their arms above their head. Spiral acquisition was performed on the entire chests, while the subject took a deep breath in and held it in apnea. All scans were obtained using a low-dose regimen, with the machine set at 120 kVp, 0.984:1 pitch ratio, 0.5 second~~s~~ rotation time and they were reconstructed with thin-slices (1-1.25mm) overlapping 0.8-mm intervals. Any further repeat scan was required to follow the same low-dose protocol. The effective radiation dose ranged from 0.3 to 1.5 mSv. The Engineering ensured quality checks of the CTs were every 3 months.

**Imaging Evaluation**

Radiologists also completed training in image quality and standardized image interpretation. All the LDCT images obtained from the baseline and follow-up scans were viewed using the Picture Archiving and Communication Systems (PACS, Kodak, CareStream as a new name ) at standard lung (1500 HU width and -650 HU level), soft tissue (350 HU width and 25 HU level), and bone (2000 HU width and 600 HU level) windows. Axial and coronal maximum intensity projection (MIP) images with slab thicknesses of 5 mm were used on workstation to detect the lung nodules and size measurement step by step. The location, dimension, consistency (solid, partly solid, or nonsolid), calcification, speculated sign, and other radiological features of each nodule were defined and recorded at baseline and repeated CT. For repeated screenings, the images were displayed side-by-side with the corresponding images obtained from baseline screening or recent screening. A second professional interpretation was performed by one of five chest radiologists (N.Wu, Y. Huang, J. Wang, S. Zhao, and W. Tang, with 40, 35, 30, 23 and 20 years of experience, respectively), all of whom had over 10 years of experience in interpreting low-dose screening CT images. Each LDCT scan was read to find any abnormalities and initially classify them.

**Management of lung nodules**

A nodule was defined as a nonlinear round opacity in I-ELCAP (http://www.ielcap.org/sites/default/files/I-ELCAP-protocol.pdf).

Management of lung nodules based on the I-ELCAP protocol 2006 (Supplementary Figure 1). Nodules were considered benign if they were classified or a hematoma. We recorded each noncalcified nodule’s location, size (length and width, in millimeters), and consistency (solid, partly solid, or nonsolid).

If the noncalcified nodules found were too small to meet the criteria for a positive result, the result was considered semi-positive, and LDCT was repeated 12 months later. If the result was positive, the type of workup depended on the diameter of the largest nodule.

At baseline screening, any suspicious malignant finding regardless of lesions’ size were recommended to consulted by multidisciplinary treatment (MDT) of screening team. Negative or small nodules (less than 5 mm solid/part-solid nodules or 8 mm nonsolid nodules) were recommended annual repeat LDCT scan. For indeterminate findings of positive results, if any pulmonary nodules larger than 15 mm, the imaging manifestations were evaluated by experienced radiologists. Antibiotics treatment and/or 1 month follow-up LDCT scan was recommended, but PET-CT scan would be recommended if the imaging finding did not entirely exclude malignant by non-inflammatory morphology as required, if it was positive or indeterminate, MDT consultation was required. If it was negative, annual repeat LDCT scan was recommended. 3 months LDCT follow-up scan was recommended if the diameter of solid/part solid nodule between 5-14 mm or 8-14 mm for nonsolid nodules. If nodule growth, MDT consult was recommended, if the nodule was stable or resolved, annual repeat LDCT scan was recommended.

The protocol of baseline screening was shown in Supplementary Figure 1~~.~~

MDT***

Annual repeat LDCT

MDT***

Annual repeat LDCT

Negative**

Positive or Indeterminate**

Negative or semi-positive*

Benign findings (calcified nodules or hamartoma)

Imaging manifestations

LDCT results

Make decision by radiologists with screening experience

experience

Any suspicious malignant finding

as+

Indeterminate finding of positive results

3 months LDCT

Stable or resolved

Growth

SN/PS 5-14mm or NS 8-14mm

All type of nodules

d ≥ 15mm

Inflammation suspected by morphology

Yes

No

Antibiotics treatment and/or 1 month f/u

PET-CT or biopsy for SN/PS

Completely or partially resolved

Stable or enlarged

*Negative: no any non-calcified pulmonary nodules were found on LDCT imaging.

Semi-positive results: at least one solid/part-solid nodules diameter less than 5 mm or non-solid nodules diameter less than 8 mm was found on baseline LDCT imaging.

** Negative: No significantly FDG uptake or lower than or equal to lung background, CT morphology was less likely to be lung cancer.

Positive or Indeterminate: FDG uptake above lung background and/or combined with CT morphology to diagnose lung cancer were uncertain.

***MDT: multidisciplinary diagnosis and treatment.
